# Supplementary material for: Smilax china L. Polysaccharide Alleviates Dextran Sulphate Sodium-Induced Colitis and Modulates the Gut Microbiota in Mice
Source: Foods. 2023 Apr 13;12(8):1632. doi: 10.3390/foods12081632 (PMC10137970; doi:10.3390/foods12081632)
Supplement: Supplementary file 1 [file foods-12-01632-s001.zip › foods-2245776-supplementary.pdf]

Supplementation information

Smilax china L. polysaccharide alleviates dextran sulphate sodium-induced colitis and modulates the gut microbiota in mice

Xin Li, Gaoxiang Qiao, Lulu Chu, Lezhen Lin and Guodong Zheng *

Jiangxi Key Laboratory of Natural Product and Functional Food, College of Food Science and Engineering, Jiangxi Agricultural University, Nanchang 330045, China

* Correspondence: zgd0428@jxau.edu.cn; Tel./Fax: +86-791-83813863

Table S1. Composition of the diets (g/kg).

Diet composition	Standard (AIN-93M)
Casein	140.0
Corn starch	465.7
Dextrin	155.0
Soybean oil	40.0
Lard	-
L-cystine	1.8
Sucrose	100.0
Choline bitartrate	2.5
Cellulose	50.0
Mineral mix AIN-93	35.0
Vitamin mix AIN-93	10.0
Total	1000

Table S2. Sequencing data summary and alpha community diversity.

	Shannon	Simpson	Ace	Chao1
NCD	4.26±0.38[a]	0.04±0.02[c]	1391.37±97.64[a]	1286.10±56.67[a]
DSS	3.78±0.24[b]	0.10±0.01[ab]	1077.70±243.56[b]	1027.00±160.45[b]
SSZ	3.96±0.45[ab]	0.06±0.06[bc]	1496.74±123.93[a]	1257.35±105.79[a]
SCP_C	3.56±0.10[b]	0.12±0.01[a]	1489.50±129.22[a]	1263.06±113.63[a]
SCP_A	4.00±0.30[ab]	0.07±0.04[bc]	1347.58±151.33[a]	1263.96±103.19[a]
SCP_N	4.34±0.24[a]	0.03±0.03[c]	1536.24±203.79[a]	1378.43±139.53[a]

Alpha diversity (Shannon, Simpson, Ace and Chao1 indices) in different groups were observed. Date represents as means ± SEM (n = 5). Different letters mean statistically significant differences $P < 0.05$ in the column. NCD, normal chow diet; DSS, dextran sodium sulfate; SSZ, sulfasalazine; SCP_C, crude polysaccharide form *Smilax china* L.; SCP_A, acidic polysaccharide form *Smilax china* L.; SCP_N, neutral polysaccharide form *Smilax china* L.

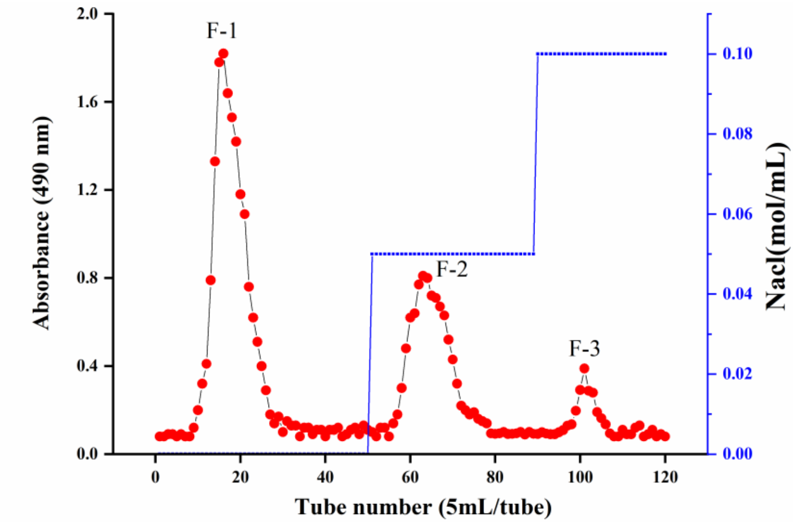

Figure S1. Elution curve of SCP_N, SCP_A on DEAE cellulose column. Purification of polysaccharide fractions. F-1, SCP_N; F-2, SCP_A; The red, blue indicate the elution curve and NaCl eluent layer, respectively.

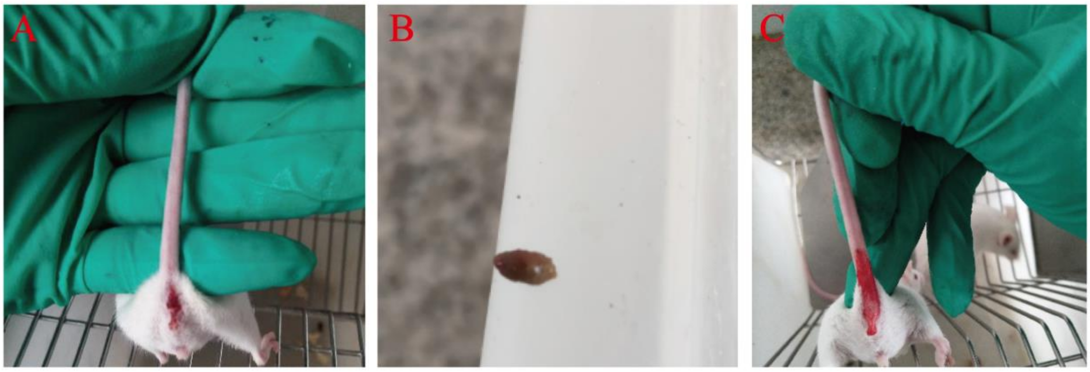

Figure S2. DSS mice with the typical symptoms of UC like diarrhea, bloody stool. (A) DSS group archorrhagia. (B) DSS group bloody stools. (C) DSS group severe bleeding from the anus.

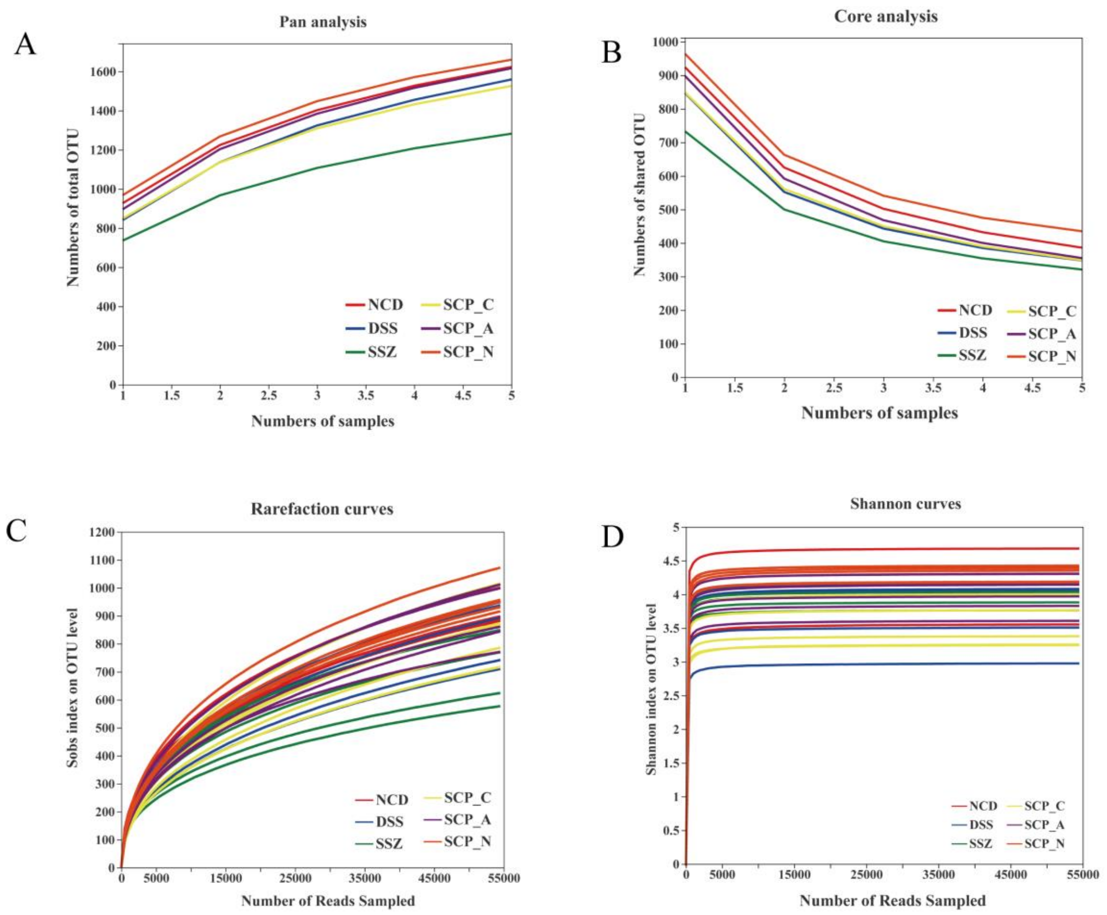

Figure S3. Alpha diversity analysis of polysaccharide-treated microbiota. (A, B) Pan/core curves (C) Rarefaction curves, and (D) Shannon index.
